# Fruit and vegetable intake and the risk of cataract: insights from the UK Biobank study

**DOI:** 10.1038/s41433-023-02498-9

**Published:** 2023-03-27

**Authors:** Huiya Fan, Xiaotong Han, Xianwen Shang, Zhuoting Zhu, Mingguang He, Guihua Xu, Zilin Chen, Ruidong Deng

**Affiliations:** 1 Department of Ophthalmology, Huizhou Central People’s Hospital, Huizhou, China; 2 State Key Laboratory of Ophthalmology, Zhongshan Ophthalmic Center, Sun Yat-sen University, Guangdong Provincial Key Laboratory of Ophthalmology and Visual Science, Guangdong Provincial Clinical Research Center for Ocular Diseases, Guangzhou, China; 3 Centre for Eye Research Australia, Royal Victorian Eye and Ear Hospital, Melbourne, VIC Australia; 4 Ophthalmology, Department of Surgery, University of Melbourne, Melbourne, VIC Australia

**Keywords:** Risk factors, Lens diseases

## Abstract

**Purpose:**

A prospective cohort study to investigate the association between fruit and vegetable (F&V) intake and the risk of cataract.

**Methods:**

We included 72,160 participants who were free of cataract at baseline from the UK Biobank. Frequency and type of F&V intake were assessed using a web-based 24 h dietary questionnaire from 2009 to 2012. Development of cataract during the follow-up was defined by self-report or hospital inpatient records up to 2021. Cox proportional regression models were used to estimate the association between F&V intake and incident cataract.

**Results:**

During a mean follow-up of 9.1 years, 5753 participants developed cataract with a corresponding incidence of 8.0%. After adjusting for multiple demographic, medical and lifestyle covariates, higher intake of F&V were associated with a lower risk of cataract (≥6.5 vs. <2 servings/week: hazards ratio [HR]: 0.82, 95% CI: 0.76 to 0.89; *P* < 0.0001). Regarding specific types, significant reduced risk of cataract was found for higher intake of legumes (*P* = 0.0016), tomatoes (≥5.2 vs. <1.8 servings/week: HR: 0.94, 95% CI: 0.88 to 1.00), and apple and pear (>7 vs. <3.5 servings/week: 0.89, 95% CI: 0.83 to 0.94; *P* < 0.0001), but not for cruciferous vegetables, green leafy vegetables, berry, citrus fruit or melon. Smokers were found to benefit more from F&V intake than former and never smokers. Men also could benefit more from higher vegetable intake than women.

**Conclusions:**

More F&V intake, especially legumes, tomatoes, apple, and pear, was associated with a lower risk of cataract in this UK Biobank cohort.

## Introduction

According to the latest Global Burden of Disease Study, cataract remains the leading cause of blindness worldwide with approximately 15.2 million cases of blindness attributable to cataract in 2020 [1]. Vision loss associated with cataract has been widely reported to increase the risk of falls, decrease cognitive function, individual independence as well as quality of life [2, 3]. With the rapid ageing of the global population (2.1 billion aged 60 years or over in 2050) [4], the accompanying burden of cataract is also on the rise.

Despite that cataract could be effectively treated by modern cataract surgery, the associated economic expenses, problems related to accessibility and possibility of surgical complications could not be neglected [5, 6]. To meet the World Health Organization (WHO) objectives of healthy ageing, more studies are needed to identify modifiable risk factors for better cataract prevention and control in our daily life.

Oxidative stress has been widely reported to play an important role in cataract pathogenesis, and previous studies had sought for potential associations between dietary factors and risk of cataract [7–9]. One meta-analysis of nine articles, mostly cross-sectional, concluded a protective effect of higher vegetables consumptions on cataract in American and European populations [10]. Another review by Sella et al. suggested that a high dietary intake of fruit and vegetables (F&V), as well as vitamins A, C, D, E and K1 may be beneficial for cataract [11]. Other research assessed the effect of a special diet pattern (e.g. vegetarian, Mediterranean diet) or the total diet antioxidant capacity on risk of cataract [12–14].

Fruit and vegetables have been a cornerstone of healthy dietary recommendations, however, only limited cohort studies exist and the association between F&V intake and risk of cataract has not been established. Additionally, to our knowledge, associations between specific types of F&V intake and cataract were not clear. To address these limitations, we aimed to elucidate the associations between F&V intake and incident cataract using the large UK Biobank cohort with a wealth of data on diet intake and medical factors along the follow-up.

## Methods

### The study population

**Fig. 1:**
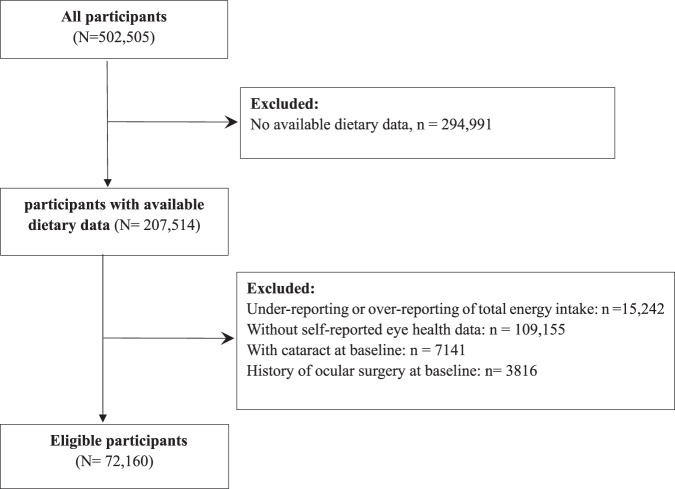
Participants selection flowchart. Study flowchart of population selection from the UK Biobank.

Participants of this study were selected from the UK Biobank, which is a large community-based cohort of over 500,000 participants from the United Kingdom [15]. Detailed study methodology has been reported previously [15]. In brief, participants aged 40 to 69 years who were registered with the National Health Service (NHS) and lived within 25 miles of any of the 22 assessment centres were invited to join the study at baseline between 2006 and 2010. The UK Biobank was conducted in accordance with the principles of the Declaration of Helsinki, and ethics approval was granted by the National Information Governance Board for Health and Social Care and the NHS North West Multicenter Research Ethics Committee (REC reference: 16/NW/0274). All participants provided informed consent through electronic signature at recruitment. The UK Biobank has been established as an open‐access resource and is globally accessible to approved researchers and scientists undertaking research to improve public health [16]. The present study was conducted under application number 62443 of the UK Biobank resource. The participants selection flowchart is shown in Fig. 1.

### Dietary intake assessment

A web-based 24 h dietary assessment tool, the validated Oxford WebQ, was used for dietary intake assessment in a subgroup of participants between 2009 and 2012 [17]. Collection of dietary data from the food frequency questionnaire in UK Biobank had been validated previously [18]. The consumption levels of F&V were categorized into five groups, with cut-off points based on the distribution of intake frequency [19]. Types of F&V intake were also linked to incident cataract. The questionnaire was administered online and only participants who finished at least one of the five questionnaires were included in the current analysis. We further excluded those without self-reported eye health data or with cataract at baseline.

The amount of each food consumed was calculated by multiplying the assigned portion size of each food by the quantity consumed. Energy intake was calculated by multiplying the quantity of consumption of each food by energy of the portion (as taken from McCance and Widdowson’s The Composition of Foods and its supplements) and then summing this across all food items [17].

Basal metabolic rate was estimated using the Henry equation [20]. Participants deemed to have under-reporting (defined as total energy intake <1.1 × basal metabolic rate) or over-reporting (defined as >2.5 × basal metabolic rate) of total energy intake were further excluded from the analysis.

### Ascertainment of cataract

Cataract during the follow-up was defined by self-report or hospital inpatient records, using codes for International Classification of Diseases (ICD) numbers of H250, H251, H252, H258, H259, H261, H262, H263, H264, H268, H269, H280, H281, H282; ICD9: 366, 3661, 3662, 3663, 3664, 3665, 3668, 3669. In addition, we used surgical procedures (OPCS4) to identify cataract events (codes: C71.2 or C75.1). The earliest recorded code date was used as the onset date of cataract. Person-years were calculated from the date of baseline assessment to the date of onset cataract, date of death, or the end of follow-up (December 31, 2020, for England and Wales and January 18, 2021, for Scotland), whichever came first.

### Covariates

Participants answered a detailed touch-screen questionnaire which included information regarding their age, gender, ethnicity, education, household income, history of disease and surgery, use of vitamin supplement (yes/no), as well as lifestyle factors, including sleep duration (hours/day), alcohol drinking (never/previous/current) and smoking status (never/former/current). Physical activity (PA) was assessed using the short form International Physical Activity Questionnaire [21], and the metabolic equivalent (MET)- minutes/week of PA was calculated based on their answers to time spent on walking, moderate PA and vigorous PA. Weight was measured with the BV-418 MA body composition analyser (Tanita), and height was measured in a barefoot standing position using a Seca 202 height measure. Body mass index was calculated based on measured weight (kg) divided by measured height (m) squared. Depression was recorded during the interview with a research nurse. Blood cholesterols, including triglycerides, high-density lipoprotein cholesterol (HDL-C) and low-density lipoprotein cholesterol (LDL-C), were measured by direct enzymatic methods ((Konelab, Thermo Fisher Scientific, Waltham, Massachusetts), and Glycosylated haemoglobin, Type A1C (HbA1c)) was measured using high-performance liquid chromatography on a Bio-Rad Variant II Turbo.

### Statistical analysis

Ethnicity was categorized into three groups (whites, non-whites and unknown) and education was categorized into four groups (0–5 years, 6–12 years, ≥13 years and missing). Household income was also divided into seven subgroups, including <18000, 18000–30999, 31000–51999, 52000–100000, >100000, unknown and not answered.

One-way ANOVA and chi-square test for categorical variables was used to examine the difference of baseline characteristics among participants with different quintiles (Q) of fruit and vegetable intake. Cox Proportional Regression models were used to estimate the risk for incident cataract associated with vegetable and fruit intake. Model 1 was adjusted for age and gender; Model 2 was adjusted for Model 1 plus ethnicity, education, household income, total energy intake, vitamin supplement intake, alcohol consumption, physical activity, smoking, and sleep duration; Model 3 was adjusted for Model 2 plus BMI, HDL-C, LDL-C, triglycerides, HbA1c, hypertension, and depression; Model 4 was adjusted for Model 3 plus vitamin D and medications for lipids, blood pressure, or glucose lowering. Cox Proportional Regression models were used to test whether the association between vegetable/fruit intake and incident cataract was moderated by age, gender, education, smoking, obesity, hypertension, diabetes, or depression. The analysis was adjusted for age, gender, ethnicity, education, household income, total energy intake, alcohol consumption, physical activity, smoking, sleep duration, BMI, HDL-C, LDL-C, triglycerides, HbA1c, hypertension, and depression. All analyses were completed using the SAS software package (version 9.4; SAS Institute, Cary, NC, USA). A two-tailed *P*-value of <0.05 was used as the level of statistical significance.

## Results

Of all 502,505 baseline participants from the UK biobank, 207,514 (41.30%) with available dietary data were included in this study. After further excluding 15,242 participants (7.35%) with under- or over reporting of total energy intake, 109,155 participants (52.60%) without self-reported eye health data, 7141 participants (3.44%) already had cataract at baseline, and 3816 participants (1.84%) with history of ocular surgery at baseline, the remaining 72,160 participants (34.77%) were included in the final analysis.

No significant difference was found for ethnicity, sleep duration, LDL-C level or diabetes status among participants in the five quintile groups of F&V intake at baseline, but significant differences were identified for all other baseline characteristics as shown in Table 1. Women, as well as participants with older age, higher education level, more PA and vitamins supplements intake tended to have more F&V intake. Participants with higher level of F&V intake also tended to have larger daily energy intake, lower BMI, lower HbA1c, lower triglycerides and higher HDL-C. The proportion of participants with hypertension and depression also decreased with more F&V intake.

**Table 1:** Baseline characteristics by quintiles of fruit and vegetable intake.

	Fruit and vegetable intake					
	Quintile 1	Quintile 2	Quintile 3	Quintile 4	Quintile 5	
Range (Servings/day)	<2	2.0–3.1	3.2–4.4	4.5–6.4	≥6.5	
Age (years)	55.39 + /8.21	55.92 + /7.97	56.32 + /7.96	56.76 + /7.80	57.08 + /7.71	<0.0001
Gender						<0.0001
Women	8458 (48.7)	6113 (53.2)	7500 (56.7)	9264 (59.6)	9444 (64.9)	
Men	8902 (51.3)	5370 (46.8)	5722 (43.3)	6275 (40.4)	5112 (35.1)	
Ethnicity						0.10
Whites	16143 (93.0)	10804 (94.1)	12467 (94.3)	14675 (94.4)	13403 (92.1)	
Non-whites	1149 (6.6)	635 (5.5)	704 (5.3)	811 (5.2)	1087 (7.5)	
Unknown	68 (0.4)	44 (0.4)	51 (0.4)	53 (0.3)	66 (0.5)	
Education						<0.0001
0–5 years	1970 (11.3)	997 (8.7)	999 (7.6)	1221 (7.9)	1222 (8.4)	
6–12 years	9269 (53.4)	5592 (48.7)	6155 (46.6)	7217 (46.4)	6832 (46.9)	
≥13 years	5988 (34.5)	4848 (42.2)	6010 (45.5)	7046 (45.3)	6421 (44.1)	
Missing	133 (0.8)	46 (0.4)	58 (0.4)	55 (0.4)	81 (0.6)	
Household income (pounds)						<0.0001
<18,000	3034 (17.5)	1602 (14.0)	1701 (12.9)	2031 (13.1)	2179 (15.0)	
18,000–30,999	3765 (21.7)	2415 (21.0)	2786 (21.1)	3381 (21.8)	3343 (23.0)	
31,000–51,999	4179 (24.1)	2882 (25.1)	3331 (25.2)	3930 (25.3)	3431 (23.6)	
52,000–100,000	3399 (19.6)	2551 (22.2)	3012 (22.8)	3499 (22.5)	2930 (20.1)	
>100,000	995 (5.7)	857 (7.5)	995 (7.5)	1083 (7.0)	959 (6.6)	
Unknown	583 (3.3)	332 (2.9)	373 (2.9)	422 (2.7)	490 (3.4)	
Not answered	1405 (8.1)	844 (7.3)	1024 (7.7)	1193 (7.7)	1224 (8.4)	
Sleep duration (hours/day)	7.09 + /1.21	7.14 + /1.08	7.15 + /1.07	7.13 + /1.09	7.09 + /1.13	0.99
Physical activity (MET minutes/week)^†^	2442.67 + /2334.90	2433.62 + /2200.33	2465.05 + /2178.11	2613.49 + /2299.46	2929.70 + /2479.56	<0.0001
Smoking						<0.0001
Never	8973 (51.7)	6502 (56.6)	7705 (58.3)	9054 (58.3)	8352 (57.4)	
Former	6116 (35.2)	3967 (34.5)	4587 (34.7)	5476 (35.2)	5284 (36.3)	
Current	2228 (12.8)	990 (8.6)	903 (6.8)	974 (6.3)	880 (6.0)	
Missing	43 (0.2)	24 (0.2)	27 (0.2)	35 (0.2)	40 (0.3)	
Alcohol consumption						0.0002
Never	603 (3.5)	355 (3.1)	455 (3.4)	519 (3.3)	647 (4.4)	
Previous	627 (3.6)	326 (2.8)	334 (2.5)	483 (3.1)	562 (3.9)	
Current	16114 (92.8)	10792 (94.0)	12423 (94.0)	14533 (93.5)	13341 (91.7)	
Missing	16 (0.1)	10 (0.1)	10 (0.1)	4 (0.0)	6 (0.0)	
Energy intake/day (KJ)	8765.55 + /2459.85	8802.01 + /2285.90	8864.03 + /2279.81	9037.80 + /2364.21	9358.29 + /2603.48	<0.0001
Vitamins supplement						<0.0001
No	12378 (71.3)	6907 (60.1)	7272 (55.0)	8032 (51.7)	7024 (48.3)	
Yes	4982 (28.7)	4576 (39.9)	5950 (45.0)	7507 (48.3)	7532 (51.7)	
BMI (kg/m^2^)	27.42 + /4.62	26.85 + /4.38	26.63 + /4.41	26.65 + /4.43	26.78 + /4.66	<0.0001
HDL-C (mmol/L)	1.44 + /0.35	1.48 + /0.36	1.50 + /0.37	1.51 + /0.36	1.52 + /0.37	<0.0001
LDL-C (mmol/L)	3.54 + /0.84	3.56 + /0.80	3.55 + /0.82	3.56 + /0.83	3.55 + /0.82	0.081
Triglycerides (mmol/L)	1.75 + /0.99	1.66 + /0.92	1.64 + /0.90	1.63 + /0.90	1.62 + /0.90	<0.0001
Glycosylated haemoglobin (mmol/mol)	36.00 + /6.13	35.72 + /5.78	35.67 + /5.76	35.77 + /5.55	35.85 + /5.66	0.028
Depression						<0.0001
No	16185 (93.2)	10905 (95.0)	12637 (95.6)	14837 (95.5)	13802 (94.8)	
Yes	1175 (6.8)	578 (5.0)	585 (4.4)	702 (4.5)	754 (5.2)	
Hypertension						<0.0001
No	12902 (74.3)	8855 (77.1)	10199 (77.1)	11973 (77.1)	11110 (76.3)	
Yes	4458 (25.7)	2628 (22.9)	3023 (22.9)	3566 (22.9)	3446 (23.7)	
Diabetes						0.075
No	16651 (95.9)	11102 (96.7)	12839 (97.1)	15006 (96.6)	14015 (96.3)	
Yes	709 (4.1)	381 (3.3)	383 (2.9)	533 (3.4)	541 (3.7)	

*BMI* body mass index, *HDL-C* low-density lipoprotein cholesterol, *HDL-C* high-density lipoprotein cholesterol.

ANOVA for continuous variables and Chi-square test for categorical variables were used to test the difference in baseline characteristics across quintiles of vegetable and fruit intake.

During a mean follow-up of 9.1 years (standard deviation = 1.5), 5753 participants developed cataract. As shown in Table 2, after adjusting for all potential covariates in this study, higher intakes of F&V were associated with a lower risk of cataract (*P* for trend <0.0001). Specifically, compared to participants with less than 2 servings/day of F&V intake, those with ≥6.5 serving/day intake had a 18% decrease (95% CI: 24% to 11%) in the risk of incident cataract during the follow-up. Taking apart, higher intake of fruit (Model 4: HR 0.77, *P* < 0.0001), but not vegetable, was associated with lower risk of cataract development during the follow-up in the trend analysis. Nevertheless, participants in the highest quintile of vegetable (HR: 0.89, 95% CI: 0.82 to 0.96) and fruit (HR: 0.77, 95% CI: 0.71 to 0.83) group demonstrated significantly decreased risk of cataract, compared to those in the lowest quintile group. Sensitivity analyses which only included participants with at least two dietary surveys (Table S1) and at least three surveys (Table S4) showed similar results.

**Table 2:** Risk for incident cataract associated with fruit and vegetable intake.

	Consumption level					*P*-value
	Quintile 1	Quintile 2	Quintile 3	Quintile 4	Quintile 5	for trend
Fruit and vegetable						
Range (servings/day)	<2	2.0–3.1	3.2–4.4	4.5–6.4	≥6.5	
Events	1425	847	1011	1266	1204	
Participants	17360	11483	13222	15539	14556	
Person-years	159591.74	104279.87	119572.91	141740.21	135029.62	
HR (95% CI), Model 1	Reference	0.86 (0.79–0.94)	0.86 (0.79–0.93)	0.86 (0.79–0.92)	0.81 (0.75–0.87)	<0.0001
HR (95% CI), Model 2	Reference	0.88 (0.80–0.95)	0.87 (0.80–0.95)	0.87 (0.81–0.94)	0.81 (0.75–0.88)	<0.0001
HR (95% CI), Model 3	Reference	0.89 (0.81–0.96)	0.88 (0.81–0.96)	0.88 (0.81–0.95)	0.82 (0.76–0.89)	<0.0001
HR (95% CI), Model 4	Reference	0.89 (0.81–0.97)	0.88 (0.81–0.96)	0.88 (0.81–0.95)	0.82 (0.76–0.89)	<0.0001
Vegetable						
Range (servings/day)	<0.8	0.8–1.6	1.7–2.5	2.6–2.9	>3.9	
Events	1166	1085	986	1308	1208	
Participants	14484	14369	12806	16055	14446	
Person-years	134980.78	130450.09	115724.19	144920.82	134138.47	
HR (95% CI), Model 1	Reference	0.90 (0.83–0.98)	0.90 (0.83–0.98)	0.91 (0.84–0.98)	0.87 (0.81–0.95)	0.015
HR (95% CI), Model 2	Reference	0.91 (0.84–0.99)	0.92 (0.85–1.00)	0.93 (0.85–1.00)	0.88 (0.81–0.95)	0.041
HR (95% CI), Model 3	Reference	0.92 (0.85–1.00)	0.93 (0.86–1.02)	0.94 (0.87–1.02)	0.89 (0.82–0.96)	0.077
HR (95% CI), Model 4	Reference	0.92 (0.85–1.00)	0.93 (0.86–1.02)	0.94 (0.87–1.02)	0.89 (0.82–0.96)	0.078
Fruit						
Range (servings/day)	0	>0, <1	1.0–1.9	2.0–2.9	≥3	
Events	1284	481	1378	1150	1460	
Participants	14473	6943	18284	14797	17663	
Person-years	131594.09	60652.46	166868.25	136378.66	164720.88	
HR (95% CI), Model 1	Reference	0.87 (0.78–0.96)	0.77 (0.71–0.83)	0.74 (0.68–0.80)	0.75 (0.69–0.81)	<0.0001
HR (95% CI), Model 2	Reference	0.90 (0.81–1.00)	0.79 (0.73–0.86)	0.76 (0.70–0.82)	0.76 (0.70–0.82)	<0.0001
HR (95% CI), Model 3	Reference	0.91 (0.82–1.01)	0.81 (0.75–0.87)	0.77 (0.71–0.84)	0.77 (0.71–0.83)	<0.0001
HR (95% CI), Model 4	Reference	0.91 (0.82–1.01)	0.81 (0.75–0.88)	0.77 (0.71–0.84)	0.77 (0.71–0.83)	<0.0001

*HR* hazard ratio, *CI* confidence interval.

Cox Proportional Regression models were used to estimate the risk for incident cataract associated with vegetable/fruit intake. Model 1 was adjusted for age and gender; Model 2 was adjusted for Model 1 plus ethnicity, education, household income, total energy intake, vitamin supplement, alcohol consumption, physical activity, smoking, and sleep duration; Model 3 was adjusted for Model 2 plus BMI, HDL-C, LDL-C, triglycerides, HbA1c, hypertension, and depression; Model 4 was adjusted for Model 3 plus vitamin D and medications for lipids, blood pressure, or glucose lowering.

By further analysing different types of vegetable intake, we found that higher intake of legumes (*P* for trend = 0.0015) was significantly associated with lower risk of cataract, while different intake of cruciferous vegetables and green leafy vegetables showed no significant impact on the cataract risk (Table 3). The highest intake group of tomatoes also demonstrated a significantly reduced risk of cataract (HR: 0.94, 95% CI: 0.88 to 1.00), compared to the lowest intake group. Regarding different types of fruit, higher intakes of apple and pear showed a significantly reduced risk of cataract (*P* for trend <0.0001; >7 vs. <3.5 servings/week: 0.88, 95% CI: 0.83 to 0.94) (Table 4). While higher intakes of berries, citrus fruit and melon showed no significant association with the risk of incident cataract. Sensitivity analyses which only included participants with at least two dietary surveys (Tables S2, 3) and at least three surveys (Tables S5, 6) showed similar results, despite that the associations between different subtypes of vegetable intake and cataract risk were no longer significant, perhaps due to a smaller sample size.

**Table 3:** Risk for incident cataract associated with intake of different types of vegetables.

	Consumption level			
	Quintile 1	Quintile 2	Quintile 3	
Cruciferous vegetables				
Range (servings/week)	<1.8	1.8–4.6	>4.6	
Events	3181	1279	1293	
Participants	41258	15961	14941	
Person-years	381500	140749.95	137964.35	
HR (95% CI), Model 1	Reference	1.05 (0.98–1.12)	0.95 (0.89–1.02)	0.060
HR (95% CI), Model 2	Reference	1.05 (0.98–1.12)	0.96 (0.90–1.03)	0.092
HR (95% CI), Model 3	Reference	1.06 (0.99–1.13)	0.97 (0.91–1.03)	0.084
HR (95% CI), Model 4	Reference	1.14 (1.07–1.22)	0.97 (0.91–1.03)	<0.0001
Green leafy vegetables				
Range (servings/week)	<1.2	1.2–3.5	>3.5	
Events	3370	815	1568	
Participants	42609	10871	18680	
Person-years	394621.4	94934.32	170658.59	
HR (95% CI), Model 1	Reference	1.00 (0.93–1.08)	0.95 (0.90–1.01)	0.25
HR (95% CI), Model 2	Reference	1.00 (0.93–1.08)	0.96 (0.90–1.02)	0.35
HR (95% CI), Model 3	Reference	1.01 (0.93–1.09)	0.96 (0.90–1.02)	0.40
HR (95% CI), Model 4	Reference	1.06 (0.99–1.13)	0.97 (0.91–1.04)	0.086
Legumes				
Range (servings/week)	<2.3	2.3–5.2	>5.2	
Events	3332	1165	1256	
Participants	41957	14627	15576	
Person-years	387458.3	128056.7	144699.33	
HR (95% CI), Model 1	Reference	1.09 (1.02–1.17)	0.96 (0.90–1.02)	0.0057
HR (95% CI), Model 2	Reference	1.10 (1.03–1.17)	0.95 (0.89–1.02)	0.0023
HR (95% CI), Model 3	Reference	1.10 (1.03–1.18)	0.95 (0.89–1.02)	0.0016
HR (95% CI), Model 4	Reference	1.10 (1.03–1.18)	0.95 (0.89–1.01)	0.0015
Tomatoes				
Range (servings/week)	<1.8	1.8–5.2	>5.2	
Events	3145	1325	1283	
Participants	39725	16876	15559	
Person-years	367159.7	150080.92	142973.73	
HR (95% CI), Model 1	Reference	1.01 (0.94–1.07)	0.92 (0.86–0.98)	0.028
HR (95% CI), Model 2	Reference	1.02 (0.95–1.09)	0.93 (0.87–1.00)	0.064
HR (95% CI), Model 3	Reference	1.02 (0.96–1.09)	0.94 (0.88–1.00)	0.066
HR (95% CI), Model 4	Reference	1.02 (0.96–1.09)	0.94 (0.88–1.00)	0.067

*HR* hazard ratio, *CI* confidence interval.

Cox Proportional Regression models were used to estimate the risk for incident cataract associated with types of vegetable intake. Model 1 was adjusted for age and gender; Model 2 was adjusted for Model 1 plus ethnicity, education, household income, total energy intake, vitamin supplement, alcohol consumption, physical activity, smoking, and sleep duration; Model 3 was adjusted for Model 2 plus BMI, HDL-C, LDL-C, triglycerides, HbA1c, hypertension, and depression; Model 4 was adjusted for Model 3 plus vitamin D and medications for lipids, blood pressure, or glucose lowering.

**Table 4:** Risk for incident cataract associated with intake of different types of fruits.

	Consumption level			
	Quintile 1	Quintile 2	Quintile 3	
Berry				
Range (servings/week)	≤1.8	>1.8		
Events	4526	1227		
Participants	56871	15289		
Person-years	523266.81	136947.54		
HR (95% CI), Model 1	Reference	0.92 (0.86–0.98)		0.0087
HR (95% CI), Model 2	Reference	0.95 (0.89–1.02)		0.13
HR (95% CI), Model 3	Reference	0.96 (0.90–1.02)		0.17
HR (95% CI), Model 4	Reference	0.96 (0.90–1.02)		0.17
Citrus fruit				
Range (servings/week)	<2.3	2.3–7.0	>7	
Events	3349	861	1543	
Participants	42516	11747	17897	
Person-years	389971.86	103745.93	166496.56	
HR (95% CI), Model 1	Reference	0.92 (0.86–0.99)	0.96 (0.90–1.02)	0.068
HR (95% CI), Model 2	Reference	0.94 (0.87–1.02)	0.97 (0.91–1.03)	0.22
HR (95% CI), Model 3	Reference	0.95 (0.88–1.03)	0.97 (0.91–1.03)	0.40
HR (95% CI), Model 4	Reference	0.95 (0.88–1.03)	0.97 (0.91–1.03)	0.36
Melon				
Range (servings/week)	0	>0		
Events	5351	402		
Participants	67406	4754		
Person-years	617952.51	42261.84		
HR (95% CI), Model 1	Reference	1.07 (0.96–1.18)		0.22
HR (95% CI), Model 2	Reference	1.08 (0.97–1.19)		0.14
HR (95% CI), Model 3	Reference	1.09 (0.98–1.20)		0.11
HR (95% CI), Model 4	Reference	1.09 (0.98–1.20)		0.11
Apple and pear				
Range (servings/week)	<3.5	3.5–7.0	>7.0	
Events	3445	666	1642	
Participants	42674	8900	20586	
Person-years	389457.97	77390.17	193366.21	
HR (95% CI), Model 1	Reference	0.99 (0.91–1.08)	0.87 (0.82–0.92)	<0.0001
HR (95% CI), Model 2	Reference	1.01 (0.93–1.10)	0.88 (0.83–0.93)	<0.0001
HR (95% CI), Model 3	Reference	1.03 (0.94–1.12)	0.89 (0.83–0.94)	<0.0001
HR (95% CI), Model 4	Reference	1.03 (0.94–1.12)	0.88 (0.83–0.94)	<0.0001

*HR* hazard ratio, *CI* confidence interval.

Cox Proportional Regression models were used to estimate the risk for incident cataract associated with vegetable/fruit intake. Model 1 was adjusted for age and gender; Model 2 was adjusted for Model 1 plus ethnicity, education, household income, total energy intake, vitamin supplement, alcohol consumption, physical activity, smoking, and sleep duration; Model 3 was adjusted for Model 2 plus BMI, HDL-C, LDL-C, triglycerides, HbA1c, hypertension, and depression; Model 4 was adjusted for Model 3 plus vitamin D and medications for lipids, blood pressure, or glucose lowering.

Moderation analysis showed that the associations between fruit and vegetable intake and cataract risk were different for participants with different smoking status (*P* for interaction = 0.0015 for F&V intake, 0.0002 for vegetable intake and 0.036 for fruit intake) (Table S7). Current smokers tended to benefit the most from more F&V intake, followed by former smokers and never smokers. In addition, men were more likely to benefit from more vegetable intake on cataract risk than women (*P* for interaction = 0.022). No significant interaction was identified for other factors including age, ethnicity, obesity, diabetes or depression (data not shown).

## Discussion

In this large-scale prospective cohort study, we found that a higher intake of F&V was associated with lower risk of incident cataract. Regarding specific types of F&V intake, a significantly reduced risk was identified for higher intakes of legumes, tomatoes, apple and pear, but not for cruciferous vegetables, green leafy vegetables, berries, citrus fruit or melons.

The associations between F&V intake and risk of cataract have been reported in several previous cohort studies. In the European Prospective Investigation into Cancer and Nutrition (EPIC-OXFORD) cohort study, Appleby et al. found that vegetarians had lower risk of cataract than meat eaters [12]. In the Women’s Health Study (WHS), Christen et al. used data from a 10-year follow up of 39876 female health professionals, found that women with highest quintiles of F&V intake had 10–15% reduced risk of cataract as compared with those in the lowest quintile [22]. On the contrary, the Japanese Public Health Center-Based Prospective (JPHC) Study, which included 32,387 men and 39,333 women aged 45–74 years and followed up for five years, found that with more vegetable intake, the risk of cataract decreased in men but increased in women [23]. Fruit intake was not associated with cataract risk in the JPHC study. In the prospective Swedish Mammography Cohort study, Rautiainen et al. found that the dietary total antioxidant capacity, which mainly came from fruits and vegetables, were inversely associated with the risk of cataract [14]. The disparity in the study findings might be due to difference in study population, definition and measurement of F&V intake, as well as covariates included in the analysis. As suggested by the latest Cataract in the Adult Eye Preferred Practice Pattern [24], there is currently insufficient evidence to support a specific diet, and a well-balanced diet rich in F&V is recommended in general. More studies, especially interventional studies on specific types of F&G intake, are needed in the future for better guidance.

Using data from the UK Biobank, we sought to assess the effect for several specific types of F&V on the risk of incident cataract. Higher intake of legumes was significantly associated with a trend of lower cataract risk in our analysis. The Blue Mountains Eye Study showed a similar finding that the highest quintile of legume consumption group had significantly lower incidence of posterior subcapsular cataract compared with the lowest quintile, but no significant trend of this association was observed across quintiles [25]. In another population-based study in China, intake of flavonoid, which is rich in legume, was inversely associated with the risk of cataract [26]. Early in 1999, Pollack et al. found a protective effect of natural tomato extract on cataract formation in rats fed with a high-galactose diet [27]. We found that a higher intake of tomatoes (>5.2 servings/week vs. <1.8 servings/week) could significantly reduce the risk of incident cataract in this large population cohort. This is to no surprise as tomatoes are well known as great natural sources for vitamins and antioxidants.

Higher intakes of cruciferous vegetables were not found to be significantly associated with the risk of incident cataract in our study. However, in the aforementioned JPHC study, the highest quintile of cruciferous vegetables was associated with a 26% decreased risk of cataract, though only in men [23]. Liu et al. suggested that sulforaphane, a sulphur-rich compound in cruciferous vegetables, could protect human lens cells against oxidative stress based on an in-vitro study [28]. Most previous studies demonstrated an inverse association between higher intake of carotene and lutein, which are abundant in green and yellow vegetables, and reduced risk of cataract [29, 30]. However, both the BMES and JPHC study, as well as the current study, found no significant association between cataract and green or yellow vegetable intake [23, 31].

The association between fruit intake and cataract risk was also controversial in literature. The beneficial effect of fruit intake on cataract had been reported in the WHS study, but not in the JPHC study [23, 25]. One in vitro study proved that the Luffa cylindrica Roem fruit extract could prevent cataract progression [32]. In our study, significant beneficial effects on cataract risk were found for higher intakes of apple and pear, which are rich in vitamins and antioxidants. It has been reported previously that bilberry could reduce the oxidative stress in the lens tissue in rats’ lenses [33], and another in vitro study also showed anti-ageing effects of four berry extracts in lens cells [34]. However, in our study, the beneficial effect of berry intake on cataract was not statistically significant, perhaps due to a limited sample size or a small quantity of daily intake. We also identified a non-significant beneficial effect of citrus fruit which warrants further research.

Our study findings support the current dietary recommendation of at least two servings of fruits and three servings of vegetables per day for adults [35, 36], and provide further evidence for the recommendation of specific type of F&V to reduce the risk of cataract development. To our knowledge, this is the largest cohort study by far demonstrating the beneficial effect of different types of fruit and vegetables on incident cataract. Other study strengths included a long follow-up time, the collection of dietary intake using a preceding 24 h questionnaire and the availability of multiple covariates including the GRS. The confounding effects of other food intake were also accounted for by adjusting the average energy intake based on the whole questionnaire. Several limitations should be also noted. First, only a third of the baseline study population were included in the current analysis, and the UK Biobank itself is a community-based study of mainly UK population, the direct generalizability of the study findings are limited. But given the large sample size and robustness of study findings during sensitivity analyses, we suggest that the study findings are likely to be applied to more general population. Second, some cataract cases may not be captured in the medical records, which may also bias the study results. Third, assessment of F&V intake in the current study only included a subset of food items, future studies are needed to better understand the effects of other types of fruit and vegetables on the risk of cataract. The change of dietary habits over time is possible but could not be assessed by the 24 h recall questionnaire in this study. Fourth, although multiple important covariates had been adjusted in our analysis, including smoking, diabetes, BMI and PA, residual confounding effect from other co-variates may still exist (e.g., hormone use, sun exposure).

In conclusion, in this large cohort study of adult population, we identified a significant benefit of higher F&V intake on reducing the risk of incident cataract. These findings underscore the need to educate both doctors and patients to pay more attention to dietary factors, and recommend F&V consumption for better cataract management.

Supplemental information is available at Eye’s website

## Summary

### What is already known on this topic

- Cataract remains the leading cause of blindness worldwide and the disease burden is projected to increasing with population growth and ageing. Oxidative stress plays an important role in cataract pathogenesis. Higher fruit and vegetable intake is recommended in general health guidelines, but the specific associations between different types of fruit and vegetable intake and risk of cataract was not established.

### What this study adds

- Based on a large sample size and a mean follow-up of 9.1 years, we provided high level of evidence that higher intake of fruit and vegetable were beneficial regarding cataract risk. In addition, we investigated specific types of fruit vegetable intake and found that higher intake of legumes, tomatoes, apple and pear were associated with a lower risk of cataract.

### How this study might affect research, practice or policy

- Our study findings provide evidence supporting more detailed clinical dietary recommendation for cataract prevention.

### Supplementary information

Table S1, Table S2, Table S3, Table S4, Table S5, Table S6, Table S7

## Data Availability

All data will be available from the corresponding author upon request.
