# Supplementary material for: Fruit and vegetable intake and the risk of cataract: insights from the UK Biobank study
Source: Eye (Lond). 2023 Mar 27;37(15):3234–42. doi: 10.1038/s41433-023-02498-9 (PMC10564725; doi:10.1038/s41433-023-02498-9)
Supplement: Supplementary file 1 — Table S1, Table S2, Table S3, Table S4, Table S5, Table S6, Table S7 [file 41433_2023_2498_MOESM1_ESM.docx]

Table S1. Risk for incident cataract associated with vegetable and fruit intake among individuals with at least two dietary surveys

|  | Consumption level | |  |  |  |
| --- | --- | --- | --- | --- | --- |
|  | Quintile 1 | Quintile 2 | Quintile 3 | Quintile 4 | Quintile 5 |
| Fruit and vegetable |  |  |  |  |  |
| Events | 752 | 566 | 655 | 678 | 666 |
| Participants | 8543 | 7715 | 8575 | 8541 | 8407 |
| Person-years | 72892.96 | 66455.07 | 74013.13 | 73744.34 | 72694.25 |
| HR (95% CI), Model 1 | Reference | 0.80(0.71-0.89) | 0.77(0.69-0.85) | 0.77(0.69-0.85) | 0.72(0.65-0.80) |
| HR (95% CI), Model 2 | Reference | 0.80(0.72-0.90) | 0.78(0.70-0.86) | 0.77(0.70-0.86) | 0.72(0.65-0.80) |
| HR (95% CI), Model 3 | Reference | 0.82(0.73-0.91) | 0.79(0.71-0.88) | 0.79(0.71-0.88) | 0.74(0.66-0.82) |
| Vegetable |  |  |  |  |  |
| Events | 716 | 558 | 637 | 738 | 668 |
| Participants | 8591 | 7457 | 8218 | 9036 | 8479 |
| Person-years | 73887.76 | 64379.2 | 70749.21 | 77636.68 | 73146.9 |
| HR (95% CI), Model 1 | Reference | 0.83(0.74-0.93) | 0.85(0.76-0.94) | 0.84(0.76-0.93) | 0.80(0.72-0.89) |
| HR (95% CI), Model 2 | Reference | 0.84(0.75-0.94) | 0.86(0.77-0.96) | 0.85(0.76-0.94) | 0.81(0.73-0.90) |
| HR (95% CI), Model 3 | Reference | 0.85(0.76-0.95) | 0.88(0.79-0.98) | 0.86(0.78-0.96) | 0.82(0.74-0.92) |
| Fruit |  |  |  |  |  |
| Events | 812 | 563 | 626 | 625 | 691 |
| Participants | 8521 | 8058 | 8267 | 8274 | 8661 |
| Person-years | 71763.06 | 69904.64 | 71389.8 | 71684.78 | 75057.48 |
| HR (95% CI), Model 1 | Reference | 0.72(0.65-0.81) | 0.70(0.63-0.78) | 0.67(0.61-0.75) | 0.69(0.62-0.76) |
| HR (95% CI), Model 2 | Reference | 0.73(0.65-0.81) | 0.71(0.64-0.79) | 0.68(0.61-0.75) | 0.69(0.62-0.77) |
| HR (95% CI), Model 3 | Reference | 0.75(0.67-0.83) | 0.73(0.66-0.81) | 0.69(0.62-0.77) | 0.71(0.64-0.79) |

Table S2. Risk for incident cataract associated with intake of different types of vegetables among individuals with at least two dietary surveys

|  | Consumption level | |  |
| --- | --- | --- | --- |
|  | Quintile 1 | Quintile 2 | Quintile 3 |
| Cruciferous vegetables |  |  |  |
| Events | 586 | 711 | 695 |
| Participants | 7992 | 8766 | 8359 |
| Person-years | 68606.55 | 75335.47 | 71989.27 |
| HR (95% CI), Model 1 | Reference | 1.07(0.96-1.19) | 1.03(0.93-1.15) |
| HR (95% CI), Model 2 | Reference | 1.07(0.96-1.20) | 1.04(0.93-1.16) |
| HR (95% CI), Model 3 | Reference | 1.07(0.96-1.19) | 1.04(0.93-1.16) |
| Green leafy vegetables |  |  |  |
| Events | 1842 | 559 | 916 |
| Participants | 23094 | 7623 | 11064 |
| Person-years | 199010.8 | 65325.14 | 95463.84 |
| HR (95% CI), Model 1 | Reference | 0.85(0.77-0.93) | 0.90(0.83-0.98) |
| HR (95% CI), Model 2 | Reference | 0.85(0.77-0.94) | 0.91(0.84-0.99) |
| HR (95% CI), Model 3 | Reference | 0.86(0.78-0.94) | 0.92(0.85-1.00) |
| Legumes |  |  |  |
| Events | 662 | 643 | 704 |
| Participants | 8423 | 8379 | 9012 |
| Person-years | 72094.31 | 72443.36 | 77530 |
| HR (95% CI), Model 1 | Reference | 0.94(0.84-1.05) | 0.97(0.87-1.08) |
| HR (95% CI), Model 2 | Reference | 0.94(0.84-1.05) | 0.96(0.86-1.07) |
| HR (95% CI), Model 3 | Reference | 0.94(0.84-1.04) | 0.95(0.86-1.06) |
| Tomatoes |  |  |  |
| Events | 505 | 833 | 652 |
| Participants | 7265 | 10129 | 8375 |
| Person-years | 62743.06 | 87334.13 | 72095.44 |
| HR (95% CI), Model 1 | Reference | 1.07(0.96-1.19) | 0.99(0.88-1.11) |
| HR (95% CI), Model 2 | Reference | 1.07(0.96-1.19) | 1.00(0.89-1.12) |
| HR (95% CI), Model 3 | Reference | 1.07(0.96-1.19) | 1.00(0.89-1.12) |

Table S3. Risk for incident cataract associated with intake of different types of fruits among individuals with at least two dietary surveys

|  | Consumption level |  |  |
| --- | --- | --- | --- |
|  | Quintile 1 | Quintile 2 | Quintile 3 |
| Berry |  |  |  |
| Events | 2608 | 709 |  |
| Participants | 32917 | 8864 |  |
| Person-years | 283320.06 | 76479.69 |  |
| HR (95% CI), Model 1 | Reference | 0.88(0.81-0.96) |  |
| HR (95% CI), Model 2 | Reference | 0.91(0.83-0.99) |  |
| HR (95% CI), Model 3 | Reference | 0.91(0.84-0.99) |  |
| Citrus fruit |  |  |  |
| Events | 2016 | 551 | 750 |
| Participants | 25010 | 8014 | 8757 |
| Person-years | 214327.69 | 69728.67 | 75743.39 |
| HR (95% CI), Model 1 | Reference | 0.76(0.69-0.84) | 0.93(0.85-1.01) |
| HR (95% CI), Model 2 | Reference | 0.76(0.69-0.84) | 0.93(0.85-1.01) |
| HR (95% CI), Model 3 | Reference | 0.77(0.70-0.85) | 0.94(0.86-1.02) |
| Melon |  |  |  |
| Events | 5351 | 402 |  |
| Participants | 67406 | 4754 |  |
| Person-years | 617952.51 | 42261.84 |  |
| HR (95% CI), Model 1 | Reference | 1.07(0.96-1.18) |  |
| HR (95% CI), Model 2 | Reference | 1.06(0.96-1.17) |  |
| HR (95% CI), Model 3 | Reference | 1.07(0.97-1.18) |  |
| Apple and pear |  |  |  |
| Events | 1973 | 636 | 708 |
| Participants | 24057 | 8569 | 9155 |
| Person-years | 206097.85 | 74288.48 | 79413.43 |
| HR (95% CI), Model 1 | Reference | 0.86(0.78-0.94) | 0.87(0.80-0.95) |
| HR (95% CI), Model 2 | Reference | 0.86(0.79-0.94) | 0.87(0.80-0.95) |
| HR (95% CI), Model 3 | Reference | 0.87(0.80-0.96) | 0.88(0.81-0.96) |

Table S4. Risk for incident cataract associated with vegetable and fruit intake among individuals with at least three dietary surveys

|  | Consumption level |  |  |  |  |
| --- | --- | --- | --- | --- | --- |
|  | Quintile 1 | Quintile 2 | Quintile 3 | Quintile 4 | Quintile 5 |
| Fruit and vegetable |  |  |  |  |  |
| Events | 523 | 378 | 410 | 399 | 426 |
| Participants | 5453 | 5111 | 5273 | 5142 | 5433 |
| Person-years | 45821.29 | 43530.91 | 44902.34 | 43884.88 | 46355.33 |
| HR (95% CI), Model 1 | Reference | 0.74(0.64-0.84) | 0.72(0.64-0.82) | 0.71(0.62-0.81) | 0.67(0.59-0.76) |
| HR (95% CI), Model 2 | Reference | 0.74(0.65-0.85) | 0.73(0.64-0.83) | 0.71(0.62-0.81) | 0.67(0.59-0.77) |
| HR (95% CI), Model 3 | Reference | 0.76(0.66-0.86) | 0.74(0.65-0.85) | 0.72(0.63-0.83) | 0.69(0.60-0.78) |
| Vegetable |  |  |  |  |  |
| Events | 467 | 408 | 403 | 446 | 412 |
| Participants | 5316 | 5243 | 5129 | 5426 | 5298 |
| Person-years | 45002.95 | 44725.77 | 43652.56 | 46036.98 | 45076.5 |
| HR (95% CI), Model 1 | Reference | 0.84(0.73-0.96) | 0.82(0.72-0.94) | 0.83(0.73-0.95) | 0.78(0.68-0.89) |
| HR (95% CI), Model 2 | Reference | 0.85(0.74-0.97) | 0.83(0.73-0.95) | 0.84(0.73-0.96) | 0.78(0.68-0.90) |
| HR (95% CI), Model 3 | Reference | 0.86(0.75-0.98) | 0.85(0.74-0.97) | 0.85(0.75-0.97) | 0.79(0.69-0.91) |
| Fruit |  |  |  |  |  |
| Events | 569 | 355 | 407 | 386 | 419 |
| Participants | 5319 | 5210 | 5292 | 5308 | 5283 |
| Person-years | 44134.57 | 44598.24 | 45133.53 | 45439.1 | 45189.31 |
| HR (95% CI), Model 1 | Reference | 0.66(0.58-0.75) | 0.66(0.58-0.75) | 0.60(0.52-0.68) | 0.63(0.56-0.72) |
| HR (95% CI), Model 2 | Reference | 0.66(0.58-0.76) | 0.67(0.59-0.76) | 0.60(0.53-0.68) | 0.63(0.56-0.72) |
| HR (95% CI), Model 3 | Reference | 0.67(0.59-0.77) | 0.68(0.60-0.78) | 0.61(0.53-0.70) | 0.65(0.57-0.74) |

Table S5. Risk for incident cataract associated with intake of different types of vegetables among individuals with at least three dietary surveys

|  | Consumption level | |  |
| --- | --- | --- | --- |
|  | Quintile 1 | Quintile 2 | Quintile 3 |
| Cruciferous vegetables |  |  |  |
| Events | 369 | 477 | 435 |
| Participants | 4902 | 5766 | 5344 |
| Person-years | 41631.7 | 48932.57 | 45476.06 |
| HR (95% CI), Model 1 | Reference | 1.07(0.94-1.23) | 1.00(0.87-1.14) |
| HR (95% CI), Model 2 | Reference | 1.07(0.93-1.23) | 1.00(0.87-1.15) |
| HR (95% CI), Model 3 | Reference | 1.07(0.93-1.22) | 1.00(0.87-1.15) |
| Green leafy vegetables |  |  |  |
| Events | 300 | 454 | 511 |
| Participants | 4204 | 6160 | 6069 |
| Person-years | 35945.82 | 52444.3 | 51506.88 |
| HR (95% CI), Model 1 | Reference | 0.96(0.83-1.11) | 1.05(0.91-1.21) |
| HR (95% CI), Model 2 | Reference | 0.96(0.83-1.11) | 1.06(0.92-1.22) |
| HR (95% CI), Model 3 | Reference | 0.96(0.83-1.11) | 1.06(0.92-1.22) |
| Legumes |  |  |  |
| Events | 257 | 529 | 479 |
| Participants | 3213 | 7156 | 5928 |
| Person-years | 27156.58 | 61149.29 | 50360.31 |
| HR (95% CI), Model 1 | Reference | 0.90(0.78-1.05) | 1.00(0.86-1.17) |
| HR (95% CI), Model 2 | Reference | 0.90(0.78-1.05) | 1.00(0.85-1.16) |
| HR (95% CI), Model 3 | Reference | 0.90(0.77-1.04) | 0.99(0.85-1.15) |
| Tomatoes |  |  |  |
| Events | 299 | 481 | 496 |
| Participants | 4146 | 5640 | 6492 |
| Person-years | 35327.19 | 48077.24 | 55253.81 |
| HR (95% CI), Model 1 | Reference | 1.09(0.94-1.26) | 0.95(0.82-1.10) |
| HR (95% CI), Model 2 | Reference | 1.09(0.94-1.26) | 0.96(0.83-1.11) |
| HR (95% CI), Model 3 | Reference | 1.09(0.94-1.26) | 0.95(0.83-1.10) |

Table S6. Risk for incident cataract associated with intake of different types of fruits among individuals with at least three dietary surveys

|  | Consumption level | |  |
| --- | --- | --- | --- |
|  | Quintile 1 | Quintile 2 | Quintile 3 |
| Berry |  |  |  |
| Events | 1684 | 452 |  |
| Participants | 20618 | 5794 |  |
| Person-years | 175013.39 | 49481.36 |  |
| HR (95% CI), Model 1 | Reference | 0.84(0.76-0.94) |  |
| HR (95% CI), Model 2 | Reference | 0.86(0.77-0.96) |  |
| HR (95% CI), Model 3 | Reference | 0.87(0.78-0.96) |  |
| Citrus fruit |  |  |  |
| Events | 1209 | 454 | 473 |
| Participants | 14408 | 6500 | 5504 |
| Person-years | 121759.12 | 55782.77 | 46952.85 |
| HR (95% CI), Model 1 | Reference | 0.76(0.68-0.84) | 0.89(0.80-0.99) |
| HR (95% CI), Model 2 | Reference | 0.76(0.68-0.84) | 0.89(0.80-0.99) |
| HR (95% CI), Model 3 | Reference | 0.77(0.69-0.85) | 0.90(0.81-1.00) |
| Melon |  |  |  |
| Events | 5351 | 402 |  |
| Participants | 67406 | 4754 |  |
| Person-years | 617952.51 | 42261.84 |  |
| HR (95% CI), Model 1 | Reference | 1.07(0.96-1.18) |  |
| HR (95% CI), Model 2 | Reference | 1.06(0.96-1.17) |  |
| HR (95% CI), Model 3 | Reference | 1.07(0.97-1.18) |  |
| Apple and pear |  |  |  |
| Events | 1175 | 543 | 418 |
| Participants | 13582 | 7484 | 5346 |
| Person-years | 114794.3 | 64047.75 | 45652.7 |
| HR (95% CI), Model 1 | Reference | 0.81(0.73-0.90) | 0.83(0.74-0.93) |
| HR (95% CI), Model 2 | Reference | 0.82(0.74-0.91) | 0.83(0.74-0.93) |
| HR (95% CI), Model 3 | Reference | 0.83(0.75-0.92) | 0.84(0.75-0.94) |

Table S7. Moderation analysis for the association between fruit/vegetable intake and incident cataract.

|  | HR (95% CI)* | P-value | P for interaction |
| --- | --- | --- | --- |
| Fruit and vegetable |  |  | 0.0015 |
| Never smokers | 0.99(0.98-1.00) | 0.068 |  |
| Former smokers | 0.97(0.96-0.99) | <0.0001 |  |
| Current smokers | 0.92(0.88-0.96) | <0.0001 |  |
| Vegetable |  |  | 0.022 |
| Female | 1.00(0.98-1.02) | 0.97 |  |
| Male | 0.97(0.95-0.99) | 0.0082 |  |
| Vegetable |  |  | 0.0002 |
| Never smokers | 1.01(0.99-1.03) | 0.29 |  |
| Former smokers | 0.98(0.96-1.00) | 0.021 |  |
| Current smokers | 0.91(0.86-0.96) | 0.0008 |  |
| Fruit |  |  | 0.036 |
| Never smokers | 0.99(0.99-1.00) | 0.15 |  |
| Former smokers | 0.99(0.98-1.00) | 0.012 |  |
| Current smokers | 0.93(0.88-0.99) | 0.031 |  |

* Cox Proportional Regression models were used to test whether the association between vegetable/fruit intake and incident cataract was moderated by age, gender, education, smoking, obesity, hypertension, diabetes, or depression. The analysis was adjusted for age, gender, ethnicity, education, household income, total energy intake, vitamin supplement, alcohol consumption, physical activity, smoking, sleep duration, BMI, HDL-C, LDL-C, triglycerides, HbA1c, hypertension, depression, vitamin D and medications for lipids, blood pressure, or glucose lowering.
